# Reducing weight and increasing physical activity in people at high risk of cardiovascular disease: a randomised controlled trial comparing the effectiveness of enhanced motivational interviewing intervention with usual care

**DOI:** 10.1136/heartjnl-2019-315656

**Published:** 2019-12-12

**Authors:** Khalida Ismail, Adam Bayley, Katherine Twist, Kurtis Stewart, Katie Ridge, Emma Britneff, Anne Greenough, Mark Ashworth, Jennifer Rundle, Derek G Cook, Peter Whincup, Janet Treasure, Paul McCrone, Kirsty Winkley, Daniel Stahl

**Affiliations:** 1 Department of Psychological Medicine, Institute of Psychiatry, Psychology and Neuroscience, King's College London, London, UK; 2 Department of Women and Children’s Health, School of Life Course Sciences, King's College London, London, UK; 3 MRC and Asthma UK Centre in Allergic Mechanisms of Asthma, London, UK; 4 School of Population Health and Environmental Sciences, King's College London, London, London, UK; 5 Division of Population Health Sciences and Education, University of London St George's Molecular and Clinical Sciences Research Institute, London, UK; 6 Department of Health Services and Population Research, Institute of Psychiatry, King's College London, London, UK; 7 Section of Eating Disorders, Institute of Psychiatry, King's College London, London, UK; 8 Florence Nightingale Faculty of Nursing, Midwifery & Palliative Care, King's College London, London, UK; 9 Department of Biostatistics and Health Informatics, Institute of Psychiatry, King's College London, London, UK

**Keywords:** cardiovascular disease, motivational interviewing, behaviour change techniques, physical activity, lifestyle intervention, weight loss, primary care

## Abstract

**Objective:**

The epidemic of obesity is contributing to the increasing prevalence of people at high risk of cardiovascular disease (CVD), negating the medical advances in reducing CVD mortality. We compared the clinical and cost-effectiveness of an intensive lifestyle intervention consisting of enhanced motivational interviewing in reducing weight and increasing physical activity for patients at high risk of CVD.

**Methods:**

A three-arm, single-blind, parallel-group randomised controlled trial was conducted in consenting primary care centres in south London. We recruited patients aged 40–74 years with a QRisk2 score ≥20.0%, which indicates the probability of having a CVD event in the next 10 years. The intervention was enhanced motivational interviewing which included additional behaviour change techniques and was delivered by health trainers in 10 sessions over 1 year, in either group (n=697) or individual (n=523) format. The third arm received usual care (UC; n=522). The primary outcomes were physical activity (mean steps/day) and weight (kg). Secondary outcomes were changes in low-density lipoprotein cholesterol and CVD risk score. We estimated the relative cost-effectiveness of each intervention.

**Results:**

At 24 months, the group and individual interventions were not more effective than UC in increasing physical activity (mean difference=70.05 steps, 95% CI −288.00 to 147.90 and mean difference=7.24 steps, 95% CI −224.01 to 238.50, respectively), reducing weight (mean difference=−0.03 kg, 95% CI −0.49 to 0.44 and mean difference=−0.42 kg, 95% CI −0.93 to 0.09, respectively) or improving any secondary outcomes. The group and individual interventions were not cost-effective at conventional thresholds.

**Conclusions:**

Enhancing motivational interviewing with additional behaviour change techniques was not effective in reducing weight or increasing physical activity in those at high CVD risk.

## Introduction

Cardiovascular disease (CVD) remains the leading cause of mortality.^1^ Reduction in levels of physical activity and rising levels of obesity are limiting the decline in CVD mortality.^2^ The most effective interventions for primary prevention of CVD in high-risk individuals remain unclear. Walking, especially with a pedometer, is promoted as a near-perfect exercise,^3 4^ but this has not been studied in high-risk CVD populations. Lowering fat and increasing fibre, fruit and vegetable intake do reduce the risk for CVD in the short-term,^5^ but evidence of long-term benefits is needed.

Psychological processes are important in initiating and maintaining change to healthier lifestyles. One approach is to use motivational interviewing, which is a collaborative, goal‐oriented behaviour change technique that encourages the language of change.^6^ The appeal of motivational interviewing is that it is brief, has a validated competency framework and can be enhanced by other behaviour change techniques (eg, goal-setting, self-monitoring and social support).^7 8^ The small number of studies assessing the effectiveness of motivational interviewing in reducing CVD risk have produced mixed results.^9^

The primary aim was to compare the effectiveness and cost-effectiveness of enhanced motivational interviewing delivered by health trainers in increasing physical activity, reducing weight in people at high risk of CVD over 24 months was greater in those who received it in a group format compared with individual format or with usual care (UC).

## Methods

### Trial design

This was a three-arm, parallel-group randomised controlled trial (RCT), called MOVE IT (MOtiVational intErviewing InTervention), for individuals at high risk for CVD using a partially clustered design followed-up at 12 and 24 months from baseline. The three arms were: enhanced motivational interviewing in a group format, enhanced motivational interviewing in an individual format and UC. As participants of the group arm, but not the other two arms, were clustered within groups, we had a partially clustered design. The protocol is published online.^10^

### Setting

General practices with list sizes >5000 patients in 12 south London boroughs (Bexley, Bromley, Croydon, Greenwich, Kingston, Lambeth, Lewisham, Merton, Richmond, Southwark, Sutton and Wandsworth) representing a varied urban, socioeconomic and ethnically diverse population of approximately 3 million.^11^

### Participants

Potentially eligible participants were identified from patient record databases and invited for screening. The inclusion criteria were: aged ≥40 and ≤74 years; CVD 10-year risk score of ≥20.0% calculated using QRisk2 (QResearch, Nottingham, UK), which is a validated predictive tool for identifying the percentage risk of having a fatal or non-fatal cardiovascular event in the next 10 years^12^; fluent in conversational English; and permanent UK residence.

The exclusion criteria were: medical diagnosis of CVD; having a pacemaker; diabetes, kidney disease, atrial fibrillation or stroke; chronic obstructive pulmonary disease; disabling neurological disorder; severe mental illness; registered blind; housebound or resident in nursing home; unable to move about independently; more than three falls in past year; pregnancy; advanced cancer; morbid obesity (body mass index (BMI) >50 kg/m^2^); participating in a weight loss programme or another participant, already randomised, in the same household.

### Randomisation and masking

Simple randomisation of participants was conducted by an independent Clinical Trials Unit (King’s College London) using computer-generated randomisation blocks. In each block, 10 subjects were randomised to group, individual or UC arms in a 4:3:3 ratio. The unequal allocation ratio ensured that the group arm had approximately 33% more patients to compensate for the loss of power from any clustering effect.

### Baseline measures

We collected sociodemographic factors, such as age, gender, self-report ethnicity, occupational status, educational attainment and marital status. Biomedical data included weight (measured in light clothing, without shoes on the class 3 Tanita SC240 digital scale), height (measured to 0.1), BMI (kg/m²), waist and hip circumferences (cm), blood pressure (BP, mm Hg), glycated haemoglobin and fasting lipids. Lifestyle data collected were alcohol intake (Alcohol Use Disorders Identification Test (AUDIT)),^13^ smoking status and physical activity (ActiGraph GT3X accelerometer, a validated tri-axial movement sensor).^14^ Self-reported depressive symptoms were collected with the Patient Health Questionnaire 9-item (PHQ-9).^15^ Participants’ postcodes were used to calculate the English Index of Multiple Deprivation 2015 score.^16^

### Usual care

For UC, this consisted of referrals to locally commissioned community-based weight loss, smoking cessation and/or exercise programmes.

### Intervention

The theoretical framework for enhanced motivational interviewing was based on social cognitive theory, and the theory of planned behaviour which states that to change behaviour, people need to form an intention (cognition).^17 18^ Intention formation is influenced by: i) expected value or positive attitude; ii) subjective norm and iii) self-efficacy.

The intervention was manualised and consisted of 10 sessions over 12 months delivered by health trainers. Participants received a workbook, key learning points for every session, action planning worksheets, case studies, self-monitoring diaries and a pedometer with instructions on its use. The intensive phase consisted of 6 weekly sessions during the first 3 months focused on physical activity and diet. The maintenance phase consisted of four sessions delivered at 3, 6, 9 and 12 months.

The training consisted of 8 weeks of didactic learning, role-playing, group exercises and case discussions using standardised materials on motivational interviewing and behaviour change techniques drawn from cognitive behaviour therapy. Each health trainer’s competency was assessed at the end of training via a knowledge test and through observing delivery of two sessions.^10^ As additional quality assurance, all sessions were audiotaped and competency was monitored and supervised weekly by the clinical psychologist. Fidelity to the manual consisted of the health trainer recording targets set and achieved per session. In the group intervention, lasting 120 min, patients were additionally encouraged to use peer support during and between sessions. Those randomised to the individual arm received the same intervention, but without peer support, in sessions lasting 40 min.

### Outcomes

The primary outcomes were physical activity (average number of steps/day) and weight (kg) and the secondary outcomes were low-density lipoprotein (LDL) cholesterol and QRisk2 score, measured at 12-month and 24-month follow-ups and adjusted for baseline values.

### Economic outcomes

The EQ-5D-3L was used to generate quality-adjusted life years (QALY). Intervention costs were calculated taking into account staff time delivering the sessions and the unit costs included overheads and on-costs and accounted for the ratio of direct-to-indirect contact time. We assumed the unit cost/hour of a National Health Service band 3 clinician at £32.40. For the group intervention, the costs were apportioned over attendees. Other service costs were measured by combining service use data from the Client Service Receipt Inventory with unit costs.^19 20^

### Statistical analysis

The primary analyses were aligned with Consolidated Standards for Reporting Trials. Baseline characteristics of those who did and did not provide follow-up data was described.

Analyses based on an intention-to-treat principle using all available outcome data were used to estimate the differences 12 and 24 months using mixed-effects adjusted for the baseline value of the outcome. In the linear mixed model ‘treatment arm’, ‘time’ (a categorical variable with two levels: 12 and 24 months), the ‘interaction between treatment arm and time’, ‘borough’, ‘ethnicity’, ‘age’, ‘gender’ and the ‘baseline values’ of the outcome variable were fixed factors.

The random parts of the models were ‘GP practice’ (patients are nested in practices) and ‘therapy group’. The study’s design was complex as it is partially clustered and cross-classified. In the preliminary analyses with blinded data, the model did not converge, therefore we removed the random effect for therapist from the analysis. In order to model the dependency of the repeated observations of the same subjects at 12 and 24 months, we model the covariance between the residuals within the lowest level group ‘patients’ to be correlated by using an unstructured covariance pattern model.

A two-tailed α of 2.5% for the two main comparisons ‘group versus UC’ and ‘individual versus UC’ was used and 97.5% CIs are presented. All secondary hypotheses were assessed on a 5% α level with 95% CIs.

Our analysis model assumes that data are missing at random with conditions for variables predictive of missingness. We compared baseline characteristics of those with and without complete physical activity and weight follow-up data. Models were rerun with predictors related to outcome missingness included as further covariates. Fourteen sensitivity analyses adjusting for the influence of missing data, protocol violations and potential model misspecifications were conducted for the primary outcomes. Group comparison of skewed data were performed using the median test. STATA V.14 was used for the primary and secondary analyses.

### Cost-effectiveness analysis

We used bootstrapping methods to estimate 95% CIs around the mean cost differences. QALYs were calculated from the EQ-5D-3L. Area under the curve methods calculated the QALY gain over the follow-up period and QALY differences were analysed controlling for baseline EQ-5D-3L score. If costs were higher for one arm compared with another and QALY gains were greater, we constructed an incremental cost-effectiveness ratio (ICER) to show the cost per extra QALY gained. Uncertainty around cost and QALY estimates was explored using cost-effectiveness planes generated from 1000 bootstrapped resamples. Finally, we generated cost-effectiveness acceptability curves, using the net-benefit approach and bootstrapping, to assess which of the three approaches was the most cost-effective. The range of values used was £0–£100 000, including the guidance-based threshold of £20 000–£30 000.^21^ Sensitivity analyses were conducted around key costs.

### Sample size

We selected a conservative difference of 0.25 pooled SD in the main outcomes, which translates to a mean clinical difference (MCD) between two groups of 675 steps/day, 1.25 kg and 0.25 mmol/L total cholesterol.^22^ We assumed an intraclass correlation coefficient of 0.05. To detect differences in our primary outcomes at a two-tailed α of 0.025 and accounting for the comparisons of ‘group versus UC’ and ‘individual versus UC’, 1420 participants were required. Assuming approximately 17% loss to follow-up, a final sample of 1704 patients was required.

### Protocol violation

On 18 October 2016, a university-wide IT network outage occurred leading to loss of 95 24 months accelerometer data files and 2651 audiotaped intervention sessions. We repeated the accelerometer data for 87 (91.6%) participants and retrieved 395 (14.9%) sessions from other computer drives.

### Role of the funding source

The funder of the study had no role in study design, data collection, analysis, interpretation or manuscript preparation. The corresponding author had full access to all the data and final responsibility for submitting for publication.

## Results

**Figure 1:**
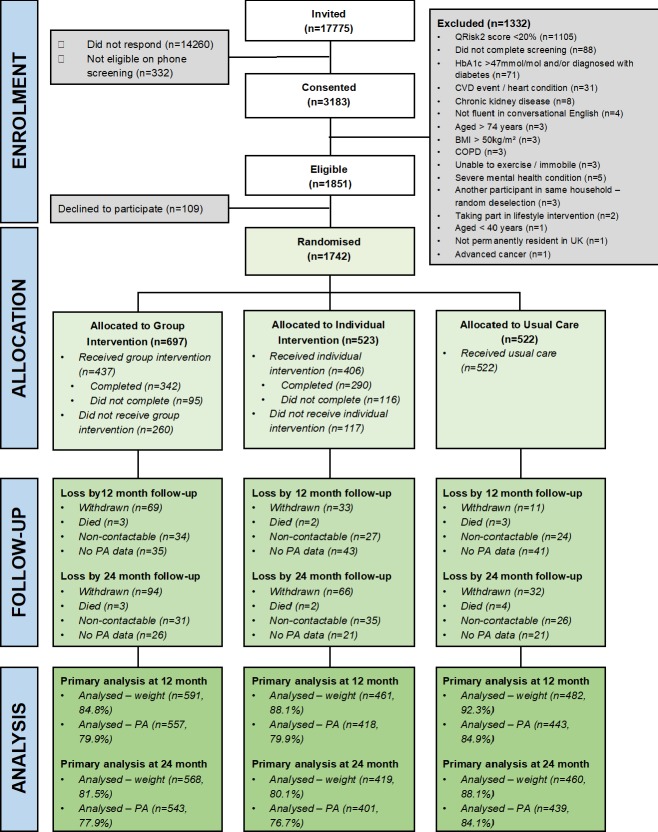
Study flow diagram. BMI, body mass index; COPD, chronic obstructive pulmonary disease; CVD, cardiovascular disease; HbA1c, glycated haemoglobin; PA. physical activity.

Of the 455 general practices invited, 135 (29.7%) consented; there was no difference in the general practices which did and did not consent.^11^ Participants were recruited between June 2013 and February 2015. Figure 1 shows the participant flow through the study. Participants were predominantly older, male and of white ethnicity; there was no significant imbalance in the baseline characteristics between arms (table 1). At baseline, participants took an average of 6757.63 (2716.55) steps/day and weighed 83.60 (15.06) kg.

**Table 1:** Baseline characteristics of participants by trial arm

	Trial arm			Total (n=1742)
	Group (n=697)	Individual (n=523)	UC (n=522)	
Age, mean (SD)	69.59 (4.16)	69.76 (4.11)	69.96 (4.05)	69.75 (4.11)
Gender, n (%)				
Male	593 (85.1)	457 (87.4)	440 (84.3)	1490 (85.5)
Female	104 (14.9)	66 (12.6)	82 (15.7)	252 (14.5)
Ethnicity, n (%)				
White	614 (88.1)	471 (90.1)	473 (90.6)	1558 (89.4)
Asian	75 (10.8)	45 (8.6)	41 (7.9)	161 (9.2)
African/Caribbean	8 (1.1)	7 (1.3)	8 (1.5)	23 (1.3)
Current employment, n (%)				
Yes	166 (23.8)	114 (21.8)	99 (19.0)	379 (21.8)
No	531 (76.2)	409 (78.2)	423 (81.0)	1363 (78.2)
Qualification, n (%)				
No formal qualifications	186 (27.2)	126 (24.4)	122 (23.8)	434 (25.3)
GCSE or equivalent	188 (27.4)	141 (27.3)	143 (27.9)	472 (27.6)
A Level or higher	311 (45.4)	249 (48.3)	247 (48.2)	807 (47.1)
Relationship status, n (%)				
Married/Cohabiting	521 (74.7)	412 (78.8)	371 (71.1)	1304 (74.9)
Divorced/Separated/ Widowed	100 (14.3)	62 (11.9)	82 (15.7)	244 (14.0)
Single	76 (10.9)	49 (9.4)	69 (13.2)	194 (11.1)
IMD 2015 quintile, n (%)				
First (most deprived)	63 (9.1)	46 (8.8)	52 (10.0)	161 (9.3)
Second	122 (17.6)	125 (23.9)	108 (20.7)	355 (20.4)
Third	136 (19.6)	88 (16.9)	93 (17.8)	317 (18.2)
Fourth	166 (23.9)	116 (22.2)	124 (23.8)	406 (23.3)
Fifth (least deprived)	208 (29.9)	147 (28.2)	145 (27.8)	500 (28.8)
Smoking status, n (%)				
Current smoker	112 (16.1)	75 (14.3)	81 (15.5)	268 (15.4)
Ex-smoker	380 (54.5)	315 (60.2)	290 (55.6)	985 (56.5)
Non-smoker	205 (29.4)	133 (25.4)	151 (28.9)	489 (28.1)
Number of cigarettes per day if current smoker, mean (SD)	11.6 (8.4)	11.0 (8.1)	11.2 (9.2)	12.7 (10.9)
Alcohol intake (AUDIT score), n (%)				
Abstainer (0)	73 (10.5)	54 (10.3)	55 (10.5)	182 (10.4)
Low risk (1-7)	506 (72.6)	397 (75.9)	383 (73.4)	1286 (73.8)
Possibly harmful (≥8)	118 (16.9)	72 (13.8)	84 (16.1)	274 (15.7)
Depressive symptoms (PHQ-9 score), mean (SD)	2.07 (3.38)	1.98 (3.05)	1.88 (3.13)	1.99 (3.21)

AUDIT, Alcohol Use Disorders Identification Test; GCSE, General Certificate of Secondary Education; IMD, Index of Multiple Deprivation; PHQ-9, Patient Health Questionnaire 9-item.

### Intervention delivery and receipt

Overall, 1220 participants were randomised to either the group or individual intervention; 28.2% did not start the intervention, 17.3% started but did not complete the intervention and 54.5% completed the intervention. The three most common reasons for participants not starting or completing the intervention were being too busy (27.4%), unable to contact (16.0%) or no longer interested in participating (15.5%). Participants in the individual arm attended more sessions (median=10, IQR=7–10) than those in the group arm (median=7, IQR=5–9; p<0.001). The online supplementary material provides further details of intervention delivery.

Fidelity to the manual was high, with the majority of participants setting targets at each session (91.6%) and the majority of these targets were achieved (90.9% achieved fully or partially; online supplementary table S3). The health trainers remained generally proficient (online supplementary material).

10.1136/heartjnl-2019-315656.supp1 Supplementary data

### Loss to follow-up

The loss to follow-up for both primary outcomes at 24 months was 18.4%, 19.7% and 11.9% for the group, individual and UC arms, respectively. The differences in loss to follow-up between the treatment arms were significant at 24 months follow-up (χ^2^(2)=13.39, p=0.001). Data were collected for 91.6% of participants for at least one of the 12-month or 24-month follow-ups, and for 79.7% of participants at both follow-ups. Participants missing 24-month outcome data walked significantly less at baseline (6376.9 (2497.2) vs 6833.1 (2752.5) steps per day, t=−2.78, p=0.006), were more likely to be current smokers (22.0% vs 14.4%, χ^2^(2)=6.4, p=0.041), to have no formal qualifications (33.2% vs 23.8%, χ^2^(2)=12.3, p=0.002) and to be more depressed (PHQ-9 score of 2.47 (3.87) vs 1.89 (3.05), t=2.44, p=0.015). There were no other baseline differences between participants with missing data compared with those with data. Online supplementary table S4 provides a breakdown of reasons for loss to follow-up.

### Primary outcomes

**Figure 2:**
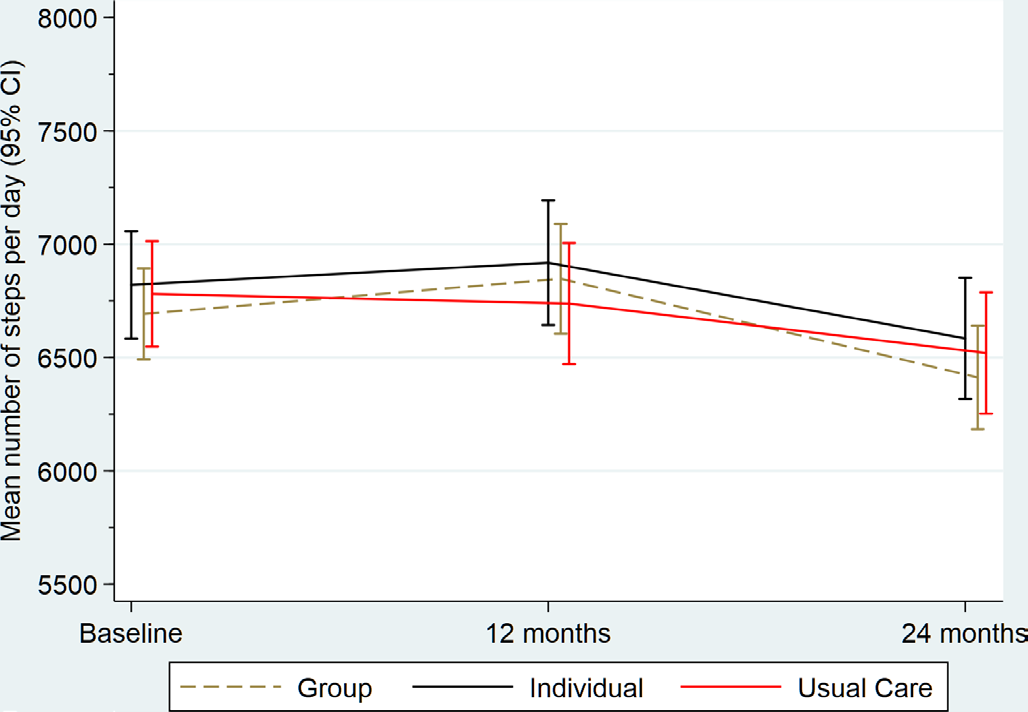
Summary of physical activity (steps) outcome by trial arm. PA, physical activity; UC, usual care.

**Figure 3:**
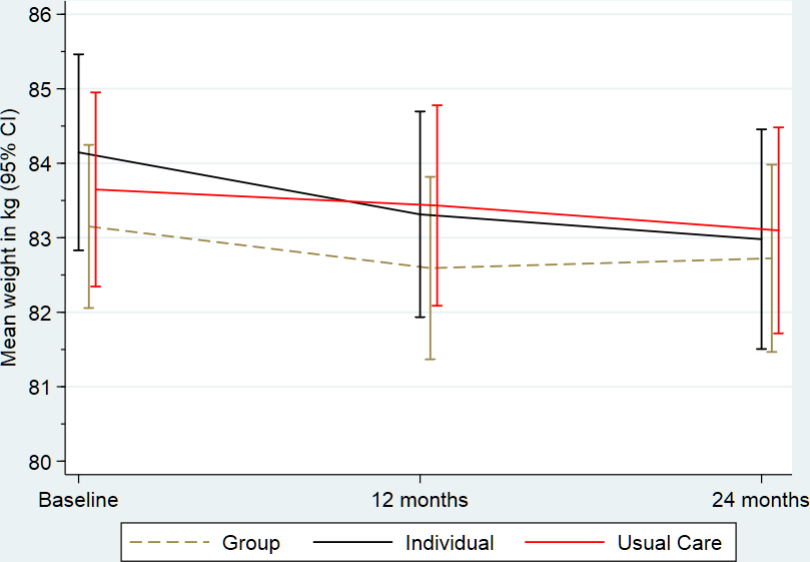
Summary of weight (kg) outcome by trial arm. UC, usual care.

Figures 2 and 3 summarise physical activity and weight, respectively, at each time point and include the pairwise comparison output. For physical activity, we did not observe any differences between the group or individual arms and UC at 12 or 24 months. For weight at 12 months, the group and individual arms had slight but significant reductions compared with UC, however, there were no differences at 24 months. All group differences (including limits of the 97.5% CIs) for physical activity and weight were below the MCD of 675 steps or 1.25 kg, respectively. Online supplementary tables S7-S8 present the fixed and random effects of the mixed-effects regression analysis on the primary outcomes. None of the sensitivity analyses altered our conclusions for either of the primary outcomes.

### Secondary outcomes

We did not observe any treatment effects for the secondary outcomes at 12 or 24 months (table 2).

**Table 2:** Descriptive summary of secondary outcomes by trial arm and time and pairwise comparison output

	Time	Trial arm			Pairwise comparisons		
		Group	Individual	UC	Group–UC	Individual–UC	Individual–group
LDL cholesterol (mmol/mol)	Baseline	3.11 (0.85)	3.14 (0.89)	3.07 (0.88)			
	12 months	2.93 (0.85)	2.92 (0.83)	2.91 (0.87)	0 (−0.07 to 0.07)	0 (−0.07 to 0.08)	0 (−0.07 to 0.08)
	24 months	3.04 (0.90)	3.02 (0.88)	2.94 (0.90)	0.07 (−0.01 to 0.15)	0.05 (−0.04 to 0.14)	−0.02 (−0.10 to 0.07)
QRisk2 score (%)	Baseline	24.95 (4.79)	25.26 (5.27)	24.93 (4.81)			
	12 months	25.18 (5.60)	25.54 (5.93)	25.50 (6.04)	−0.28 (−0.79 to 0.23)	−0.14 (−0.68 to 0.40)	0.14 (−0.36 to 0.64)
	24 months	26.73 (7.12)	27.04 (6.59)	26.69 (6.76)	0.01 (−0.68 to 0.71)	0.05 (−0.63 to 0.72)	0.03 (−0.64 to 0.71)

Cell values are mean (SD) or estimate (95% CI).

LDL, low-density lipoprotein; UC, usual care.

### Cost-effectiveness

Service costs (including zero costs for non-users) were similar for inpatient care, outpatient attendances and community contacts were similar between arms (online supplementary table S15). The intervention cost was highest for those in the individual arm. After controlling for baseline costs, total costs did not differ between the three arms. Mean EQ-5D tariff scores were similar for each arm and did not change markedly over time (online supplementary table S16). Controlling for baseline utility, QALYs did not differ between the three arms.

The group arm was less effective than UC and more expensive; as such it was dominated. Individual was more expensive and more effective. The ICER was £55 313 per QALY (£354 divided by 0.0064 QALYs). The ICER of the individual arm compared with the group arm was £8287 per QALY (£179 divided by 0.0216 QALYs) (online supplementary figures S2-S3). At a value of £30 000, the individual, group and UC arms had a 38.1%, 3.2% and 58.7% likelihood of being the most cost-effective option, respectively. The results of the sensitivity analyses did not alter the above results (online supplementary material).

### Adverse events

Five hundred twenty-three adverse events (AEs) were reported between baseline and 24-month follow-up. There were no differences in mean (SD) number of AEs experienced by participants in the group, individual and UC arms (0.37 (0.61), 0.33 (0.54) and 0.35 (0.57), respectively, (*F*(2)=0.68, p=0.51)) or in the number of fatal or non-fatal cardiovascular events (online supplementary table S17).

## Discussion

### Summary of the clinical effectiveness of MOVE IT

Enhanced motivational interviewing was not effective or cost-effective in improving physical activity, weight, LDL cholesterol or QRisk2 scores in adults at high risk of CVD over 24 months compared with UC.

### Strengths and limitations

This RCT was powered to detect small effect sizes in a real-world setting. We developed a standardised health trainer manual for replicability. We used accelerometers as the gold standard for objectively measuring physical activity.

The QRisk2 had a high false-positive rate (figure 1) because the medical records required for its algorithm were not always accurate resulting in high levels of ineligibility. Our sample may not have represented all those at higher CVD risk as the average QRisk2 score was at the lower end.^23^ We also found that boroughs with higher levels of socioeconomic deprivation and greater ethnic diversity had less uptake.^11^ Logistical barriers led to supply lag of some health trainers, which contributed to longer waiting time than desired and may explain some of the reduced uptake of the interventions. Attrition from psychological interventions is a common phenomenon,^24^ perhaps more in those allocated to groups which may induce social avoidance in some participants. Loss to follow-up was unusually lower in UC compared with the intervention arms; we have speculated that study fatigue may have contributed.

### Interpretation

One explanation for the negative finding is that our sample was skewed to non-modifiable risk factors in the QRisk2 algorithm (age, gender and ethnicity), and on average not obese with a lower than expected CVD risk score.^25^ Furthermore, participants had average and possibly optimum baseline step in line with healthy older adults.^4 26^ It may have been more appropriate to recruit by raised BMI, blood pressure and LDL cholesterol, which are modifiable risk factors,^9^ or selected a higher QRisk2 score.

A second potential explanation is that the intervention potency was subtherapeutic. For this clinical group, namely patients with a high CVD risk but with few psychological or physical symptomatic distress, the motivational interviewing approach is inappropriate. For example, the prevalence for significant depressive symptoms was very low (1.4%), lower than the general population.

Landmark studies have repeatedly shown that intensive lifestyle instruction, such as the diabetes prevention studies^27^ and weight reduction programmes,^28^ do lead to significantly improved outcomes. In these interventions, the clinically active ingredients included intensive, highly structured, prescribed dietary and/or physical activity programmes following a counselling approach and greater emphasis on formal social support, information giving and monitoring of weight and exercise. Our intervention did not prescribe a dietary or physical activity programme but aimed to address cognitions that resisted dietary and/or physical activity changes and to increase an individual’s intentions to change.

### Research implications and future directions

First, this intervention might have been more successful in those with modifiable CVD risk factors rather than the QRisk2 score alone and if we had much shorter waiting lists. The potential of an enhanced motivational interviewing approach to a younger population, those living in deprived areas and of non-white ethnicity remains unknown. We used the same strategies for recruitment regardless of socioeconomic, ethnicity and inner city status and future studies could instead oversample in these subgroups to recruit those at higher CVD risk.

Second, we may need to consider more intensive approaches to supporting lifestyle change in those most at risk of CVD. For instance, psychological constructs such as optimistic bias and habit formation are common patterns.^9 29 30^ Prioritising public health or community interventions that aim to overcome the stigma towards obesity and challenging unhelpful beliefs such as optimistic bias (“it’s not going to happen to me”) and habit formation maybe more effective than individualised approaches.

### Summary

An intensive lifestyle intervention using enhanced motivational interviewing skills was not associated with reduced weight or increased physical activity in people at high risk of CVD. Future interventions should focus on those at very high CVD risk and/or with modifiable risk factors.

### Patient and public involvement

The expert-by-experience patient participants (Jennifer Bostock, Carole Haynes) contributed to the design, conduct, reporting, and dissemination of our research.

**Key messages**

**What is already known on this subject?**

- Although mortality from cardiovascular disease (CVD) is falling, the epidemic of obesity and unhealthy lifestyles is increasing the risk of CVD.
- There is little evidence as to whether a primarily psychological approach that aims to change a person’s intentions (cognitions) can alter lifestyle behaviour.

**What might this study add?**

- This study was a response to a National Institute of Health Research commissioned brief to test whether enhanced motivational interviewing (a low-intensity psychological intervention combining motivational interviewing with additional behaviour change techniques) delivered by health trainers could improve lifestyles and thus reduce CVD risk.
- We found that enhanced motivational interviewing did not lead to a reduction in weight or increase in physical activity compared with usual care.

**How might this impact on clinical practice?**

- We conclude that enhanced motivational interviewing have little impact for reducing CVD risk in people at high CVD risk when applied to the general population.
- This raises the question as to whether the administration of low intensity psychological techniques in lifestyle-related interventions are of any clinical benefit.
