# Supplementary material for: Reducing weight and increasing physical activity in people at high risk of cardiovascular disease: a randomised controlled trial comparing the effectiveness of enhanced motivational interviewing intervention with usual care
Source: Heart. 2019 Dec 12;106(6):447–54. doi: 10.1136/heartjnl-2019-315656 (PMC7057797; doi:10.1136/heartjnl-2019-315656)
Supplement: Supplementary data [file heartjnl-2019-315656supp001.pdf]

SUPPLEMENTARY MATERIAL

Supplement to: A randomised controlled trial comparing the effectiveness of an enhanced MOtiVational intErviewing InTervention (MOVE IT) with usual care for reducing weight and increasing physical activity in people at high risk of cardiovascular disease, K Ismail et al.

Intervention delivery and receipt

Sessions received

A summary of the intervention received by participants randomised to either intervention arm is presented in Table S1. Overall, 665 (54.5%) participants completed the intervention, whereas 344 (28.2%) did not start intervention sessions and 211 (17.3%) started but did not complete intervention sessions. Differences in intervention receipt were significantly different between the intervention arms ($\chi^2(2)=38.30$, $p<0.001$).

Table S1. Summary of intervention receipt.

	Participants randomised to intervention arms					
	Group (n=697)		Individual (n=523)		Total (n=1220)	
Intervention received	n	%	n	%	n	%
**Did not start intervention**	260	37.3	117	22.4	377	30.9
**Partially completed intervention[a]**	95	13.6	116	22.2	211	17.3
**Completed intervention**	342	49.1	290	55.4	632	51.8

[a]Participants in this category completed at least one session and no more than 10, including the introductory session.

For all participants who started the intervention (n=843), a mean of 8.2 (3.2) sessions (median=9, IQR=7-11), including the introductory session, were received. The differences (tested with Mood's median test) in attendance between treatment arms is given in Table S2, with the individual arm attending significantly more sessions ($\chi^2(1)=85.71$, $p<0.001$).

Participants attended a mean of 4.8 (1.9; median=6, IQR=4-6) of the 6 intensive sessions, and 2.4 (1.7; median=3, IQR=0.5-4) of the four maintenance sessions. Participants in the individual arm attended significantly more maintenance sessions ($\chi^2(1)=95.51$, $p<0.001$) than participants in the group arm, but there was no difference for intensive sessions ($\chi^2(1)=1.14$, $p=0.29$). A comparison of attendance between the arms and session types is also presented in Table S2.

**Table S2. Number of sessions attended by participants in the intervention arms who started the intervention, and by session type**				
	Group (n=437)		Individual (n=406)	
	Mean (SD)	Median (IQR)	Mean (SD)	Median (IQR)
All session types				
Including Session 0	7.62 (2.96)	8 (6-10)	8.82 (3.38)	11 (8-11)
Excluding Session 0	6.72 (2.93)	7 (5-9)	7.83 (3.39)	10 (7-10)
Intensive sessions (n=6)	4.58 (1.80)	5 (4-6)	5.07 (1.93)	6 (6-6)
Maintenance sessions (n=4)	2.14 (1.56)	3 (0-4)	2.76 (1.70)	4 (1-4)

Time to intervention commencement

There were unexpected delays to intervention commencement. Date of intervention commencement was missing for 35 (2.9%) participants. We endeavored to start the intervention within six months of randomisation, and this was possible for 986 (83.2% of 1185) participants: 561 (82.3% of 682) in the group arm and 425 (84.5% of 503) in the individual arm. The time between randomisation and intervention start for participants randomised to each intervention arm is illustrated in Figure S1. Participants randomised to receive the intervention (group or individual arms) waited a mean of 3.12±2.70 months (min=0.16, max=15.21, median=2.07, IQR=0.92-4.99) from randomisation to their scheduled introductory session. The wait time did not differ between the group (3.17±2.78 months, n=682) and individual arms (3.04±2.59 months, n=503; t(1120.5)=0.83, p=0.41).

We investigated the association between delays to intervention commencement and the number of sessions attended. We observed a moderate negative correlation in the group arm (r(680)=-0.20, p<0.001), but not in the individual arm (r(501)=-0.03, p=0.57). The difference in correlations between the arms was significant (z=2.94, p=0.003).

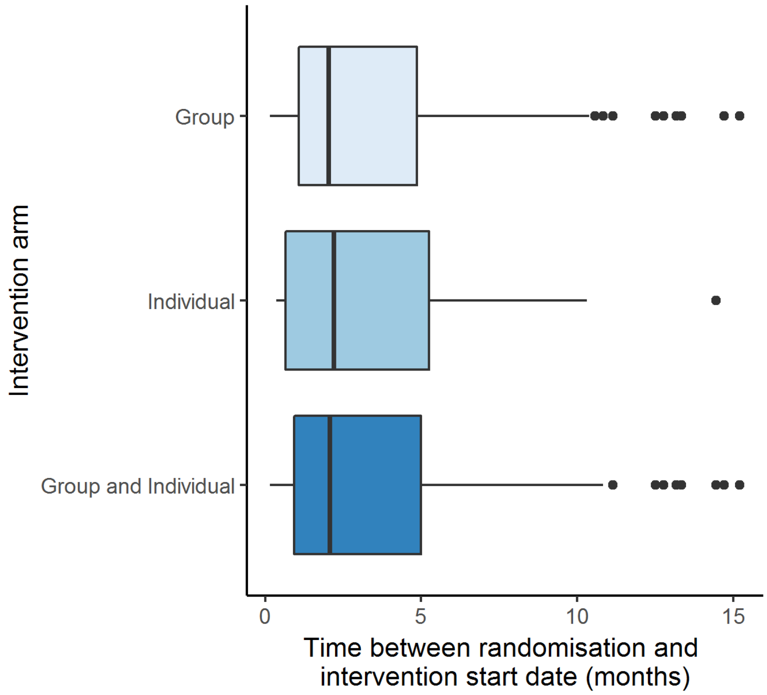

Figure S1. Time between randomisation and intervention commencement (session 0) by intervention arm.

Intervention duration

For those participants attending at least one of Sessions 1-10 (n=782), we calculated the time between Session 0 and the final session attended. Across intervention arms, the mean time between Session 0 and the final session was 330.6±173.8 days (min=6, max=808, median=366, IQR=231-440; dates were unavailable for one patient (0.1%)). Time between Session 0 and final session was significantly longer in the individual arm (355.6±173.1 days, n=373) than in the group arm (307.8±171.4 days, n=408; $t(771.4)=3.87$, $p<0.001$).

Participant adherence to intervention

After the completion of each intervention session, the health trainer was responsible for recording: (a) if the patient set a target for that session (yes/no); and (b) if that target was achieved (coded as no/partially/fully). Session 0 was not included as participants were not asked to set goals for that session.

Of the 1220 participants randomized to the intervention arms, 774 had adherence data available (63.44%). Of those, 407 (58.39% of those randomized) were in the group arm and 367 (70.17% of those randomized) in the individual arm.

The 774 participants attended a total of 5932 sessions. Table S3 shows the rate of targets set overall and by intervention arm. The overall rate of targets set differed significantly between arms ($\chi^2(1)=6.12$, p=0.01) but the distribution of targets set at each session did not differ significantly between arms ($\chi^2(9)=16.18$, p=0.06; data are not shown).

Table S3. Summary of intervention adherence: Targets set and achieved by intervention arm.

	Participants randomised to intervention arms with available data					
	Group (n=407)		Individual (n=367)		Total (n=774)	
	n	%	n	%	n	%
No target set	266	9.34	232	7.52	498	8.40
Target set at session	2582	90.66	2852	92.48	5434	91.60
Target achieved						
Not at all	143	5.54	158	5.54	301	5.54
Partially	664	25.72	679	23.81	1343	24.71
Fully	1376	53.29	1676	58.77	3052	56.16
Missing rating	399	15.45	339	11.89	738	13.58
**Total**	**2848**		**3084**		**5932**	

For each target set, the health trainer also recorded if the participant achieved it; achievement was rated as ‘not at all’, ‘partially’, or ‘fully’. Table S3 shows the number of targets achieved by intervention arm and in total; the distribution of targets achieved (excluding missing data) differed significantly between arms ($\chi^2(2)=7.25$, p=0.03). The rate of target achievement differed by session number ($\chi^2(18)=116.99$, p<0.001; data are not shown).

For the MI components for the health trainers, the average MITI scores for the spirit and reflection-to-question domains exceeded proficiency thresholds (3.5 (0.7) and 1.3 (0.9), respectively) and scores for the other three domains were slightly below the proficiency

thresholds (percent MI-adherent=84.5 (22.7), percent open questions=44.4 (14.8), percent complex reflections=37.1 (16.5)).

Loss to follow-up

Participants were not seen at follow-up for one of three reasons: (i) they had withdrawn from the study, (ii) they had died, or (iii) they were non-contactable at follow-up due date (attempts were made to contact the participant up to six months after the due date). Participants randomised to either of the intervention arms but who did not take part in the intervention did not necessarily withdraw from the study, and their data are included at follow-up to carry out the ITT analysis. Table S4 shows loss to follow-up at both 12- and 24-month follow-ups and the reasons for withdrawal given. Those who were permanently lost to follow-up at 12 months (withdrawn/died) were not contacted again at 24 months, whereas those who were non-contactable at 12 months were contacted again at 24 months.

Table S4. Reasons for loss to follow-up by arm at 12 and 24 months

	Trial arm					
	Group		Individual		UC	
	n	%	*n*	%	*n*	%
**By 12-month follow-up**						
Death	3	2.8	2	3.2	3	7.9
Withdrawal reasons:						
Ill health	11	10.4	5	8.1	3	7.9
Too busy	9	8.5	10	16.1	1	2.6
Unhappy with study	6	5.7	5	8.1	1	2.6
No perceived benefit	6	5.7	6	9.7	1	2.6
No longer interested	11	10.5	2	3.2	3	7.9
Relocated	8	7.5	2	3.2	2	5.3
Unknown	18	17.0	3	4.8	0	0.0
Non-contactable	34	32.1	27	43.5	24	63.2
Total	***106***	***100.0***	***62***	***100.0***	***38***	***100.0***
**By 24-month follow-up**						
Death	3	2.3	2	1.9	4	6.5
Withdrawal reasons:						
Ill health	22	17.2	13	12.6	15	24.2
Too busy	12	9.4	15	14.6	2	3.2
Unhappy with study	8	6.3	9	8.7	5	8.1
No perceived benefit	8	6.3	8	7.8	1	1.6
No longer interested	14	10.9	10	9.7	6	9.7
Relocated	12	9.4	3	2.9	3	4.8
Unknown	18	14.1	8	7.8	0	0.0

Non-contactable	31	24.2	35	34.0	26	41.9
Total	***128***	***100.0***	***103***	***100.0***	***62***	***100.0***

We exceeded the target follow-up of 83% required by the original sample size calculation. The total loss to follow-up across all arms of the study at 12 months was 11.8%; 15.2% for the group arm, 11.9% for the individual intervention arm and 7.3% for the UC arm. The total loss to follow-up across all arms of the study at 24 months was 16.8%; 18.4% for the group arm, 19.7% for the individual arm and 11.9% for the UC arm. Data were collected for 91.6% of all participants for at least one of 12- or 24-month follow-up, and for 79.7% of participants at both 12 and 24 months. The differences in loss to follow-up between the treatment arms were significant at 12 month ($\chi^2(2)=17.99$, $p<0.001$) and 24 month follow-up ($\chi^2(2)=13.39$, $p=0.001$). Withdrawal rates differed significantly between treatment arms at both 12- ($\chi^2(2)=27.08$, $p<0.001$) and 24-month follow-up ($\chi^2(2)=15.98$, $p<0.001$).

Accelerometer data completeness

The remaining participants did attend follow-up appointments but did not necessarily complete accelerometer wear for collection of PA outcomes. Table S5 provides details on the amount of accelerometer data collected by trial arm at each time point. The required PA data at baseline was at least five days of ≥540 minutes accelerometer wear. A number of participants did not wear the accelerometer for the sufficient amount of time at baseline and, therefore, were randomised in error. In the original analysis plan we stated that we included those with at least four days data in the primary analysis to limit exclusions. However, after discussions with the statistician of the TSC, we included all participants to minimize loss of power and to avoid inducing a potential bias. In agreement with the TSC, we used for all time points the wearable data points if participants wore the accelerometer at least one full day (≥540 minutes). Otherwise, the data were considered missing.

The total missing activity data across all arms of the study at baseline was 0.5% (n=8) and at 12 months it was 18.6%: 20.1% for the group arm, 20.1% for the individual arm and15.1% for the UC arm, $\chi^2(2)=5.9$, $p=0.052$. The total missing activity data across all arms of the study at 24 months was 20.6%: 22.1% for the group arm, 23.3% for the individual arm and 15.9% for the UC arm, $\chi^2(2)=10.4$, $p=0.006$. At least one activity follow-up measurement was available for

88.1% of the study participants: 86.4% for the group arm, 86.6% for the individual arm and 91.8% for the UC arm, $\chi^2(2)=9.7$, p=0.008.

**Table S5. Summary of accelerometer data collection at each time point and by trial arm**									
	Baseline			12-month follow-up			24-month follow-up		
Accelerometer data collection	Group arm	Individual arm	UC arm	Group arm	Individual arm	UC arm	Group arm	Individual arm	UC arm
No accelerometer issued:									
Withdrawn	-	-	-	69	33	11	94	66	32
Died	-	-	-	3	2	3	3	2	4
Non-contactable	-	-	-	34	27	24	31	35	26
Number of valid days (≥540 minutes) worn:									
0	9	7	5	35	43	41	26	21	21
1	4	3	4	2	4	3	2	3	5
2	6	7	8	8	5	7	10	8	8
3	12	3	8	12	4	9	11	8	9
4	21	16	25	20	13	17	20	10	18
5	63	46	28	47	31	40	44	40	43
6	122	87	94	115	65	84	98	70	77
7	460	354	350	353	296	283	358	262	279
Data reported are n									

Missing activity days

Because activity may depend on day of the week, the mean of less than seven activity days may be biased and we imputed intermittent missing values using the Expectation Maximization (EM) algorithm, as recommended by Catellier et al.[1] to impute missing daily values. The mean of the

observed and imputed data was used for the primary analyses. The single imputation method treats imputed values as if they were observed and hence ignores the uncertainty of the correct value. However, Catellier et al. demonstrated that EM imputation methods with intermittent missing activity data produced similar results as multiple imputation, as simulation studies showed that there were no differences in estimates of means and standard deviations. Both methods produced unbiased under missing completely at random conditions (with EM imputation sometimes performing slightly better) and hence their use is regarded as standard for intermittent missing days of activity data.[2,3] Day of the week, month, and year were included as indicator variables in the imputation model. Activity data will be imputed within each treatment arm separately. Standard clinical and demographic variables were included as potential predictors.

Predictors of missing outcome data at 12 and 24 months

In addition to missing the follow-up assessment, activity data were also missing because accelerometers were not worn sufficiently long enough. Two patients did not provide weight data at 24 months. An analysis of missing outcome data was therefore done in addition to missing attendance. A stepwise logistic regression with baseline variables, treatment arm, borough at baseline and potential predictors of missing outcome data at 12 and 24 months for weight and PA separately revealed that education, smoking status, PHQ-9 depression score and treatment arm are the most important predictors. However, only little variation was explained by the models (<2.5% pseudo-R^2). Education, smoking status, and PHQ-9 depression score were included as predictors of missingness in a sensitivity analyses to assess potential bias due to missing outcome data.

Table S6 presents baseline characteristics of participants with missing data at 12-month follow-up and those who attended 12-month follow-up and participants with missing data at 24-month follow-up and those who attended 24-month follow-up.

Table S6. Summary of baseline characteristics of participants with present versus missing primary outcome data at 12- and 24-month follow-ups.

		Weight (kg)	BMI (kg/m^2)	PA (steps)	Age (years)	QRisk2 score (%)	PHQ-9 total score
**12 months**							

Missing	*Mean*	84.07	28.89	6509.043	68.94	24.82	2.63
	SD	18.45	5.47	2024.38	4.10	5.67	3.79
	N	208	208	202	208	208	208
Present	*Mean*	83.61	28.32	6802.64	70.04	24.94	1.82
	SD	14.91	4.51	2755.58	4.04	4.74	3.07
	N	1534	1534	1532	1534	1534	1534
**24 months**							
Missing	*Mean*	84.43	28.63	6376.94	69.71	25.32	2.47
	SD	15.35	4.44	2497.19	4.32	5.12	3.87
	N	293	293	287	293	293	293
Present	*Mean*	83.43	28.20	6833.13	69.76	24.98	1.89
	SD	15.00	4.28	2752.55	4.07	4.91	3.05
	N	1449	1449	1447	1449	1449	1449

Primary outcomes

Table S7. Fixed effects of treatment on PA (steps) and weight (kg).

	PA		***Weight***	
	Estimate (95% CI)	p	Estimate (95% CI)	p
Arm				
Individual	0		0	
Group	-79.12 (-315.98–157.73)	0.51	0.03 (-0.37 – 0.43)	0.89
UC	-210.22 (-439.91–19.46)	0.073	0.55 (0.14 – 0.95)	0.08
Time				
12 month	0		0.37 (-0.01 – 0.74)	0.056
24 month	-400.7 (-585.32–-216.08)	<0.0001	-0.12 (-0.51 – 0.26)	0.53
Arm x Time				
Individual x 12 month	0		0	
Group x 24 month	1.83 (-237.16 – 240.82)	0.99	0.37 (-0.01 – 0.74)	0.056
UC x 24 months	202.98 (-31.05 – 437.0)	0.089	-0.12 (-0.51 – 0.26)	0.53
Baseline PA (centered)	0.81 (0.78 - 0.85)	<0.0001	0.98 (0.97–0.99)	<0.0001
Baseline PA (centered) x time)				
12 month	0		0	
24 month	-0.05 (-0.07 - -0.02)	0.002	0.003 (-0.007 – 0.013)	0.99
Gender, female	-416.09 (-657.24–-174.94)	0.001	-0.12 (-0.59–0.35)	0.63
Age (centered)	-34.2 (-54.49–-13.91)	0.001	-0.04 (-0.08–0.00)	0.052

Baseline borough				
Bexley	0		0	
Bromley	-17.64 (-386.86–351.59)	0.923	-0.51 (-1.21–0.19)	0.16
Lewisham	-106.37 (-524.07–311.32)	0.62	-0.33 (-1.13–0.47)	0.42
Southwark	150.07 (-354.48–654.61)	0.56	-0.94 (-1.9–0.01)	0.052
Croydon	-136.7 (-533.47–260.07)	0.50	-0.35 (-1.11–0.41)	0.36
Richmond	-27.21 (-590.03–535.62)	0.93	-0.8 (-1.89–0.29)	0.15
Sutton & Merton	-27.63 (-432.61–377.35)	0.89	-0.5 (-1.28–0.27)	0.20
Kingston	155.58 (-398.23–709.38)	0.58	-1.03 (-2.1–0.05)	0.06
Wandsworth	376.07 (-177.17–929.3)	0.18	-1.31 (-2.39–-0.23)	0.018
Lambeth	-85.87 (-560.67–388.94)	0.72	-0.76 (-1.68–0.16)	0.11
Greenwich	-21.75 (-495.5–452)	0.93	-0.26 (-1.17–0.66)	0.58
Constant	6941.9 (6566.62–7317.18)	<0.0001	83.43 (82.74–84.12)	<0.0001

Table S8. Random effects output of treatment on PA (steps) and weight (kg).

	PA	***Weight***
	Estimate (95% CI)	Estimate (95% CI)
GP	0	0
Residual variance, unstructured, by arm		
Individual: 12 month	3429521 (2994365–3927915)	10.68 (9.21–12.38)
Individual: 24 month	3162542 (2751259–3635308)	14.94 (12.98–17.19)
Individual: Covariance between 12 and 24 month	1570930 (1210002–1931859)	8.66 (7.01–10.3)
Group: 12 month	3709290 (3295446–4175105)	11.38 (10.13–12.77)
Group: 24 month	3423736 (3038490–3857827)	13.27 (11.76–14.96)
Group: Covariance between 12 and 24 month	2004068 (1656551–2351584)	7.73 (6.53–8.93)
UC: 12 month	2566291 (2248965–2928391)	9.72 (8.3–11.4)
UC: 24 month	2806634 (2458728–3203767)	16.14 (13.69–19.03)
UC: Covariance between 12 and 24 month	1559003 (1269564–1848442)	8.74 (6.88–10.61)

Sensitivity Analyses

Sensitivity analysis adjusting for imbalances on baseline characteristics

No imbalances were observed on any of the pre-specified baseline characteristics, therefore, a sensitivity analysis adjusting for these variables was not done for either of the primary outcomes. A total of 14 sensitivity analyses were conducted for each (except for two occasions) of the primary outcomes:

1. Adjusting for partially nested random effect for therapist.
2. Adding therapist and general practice as random factors and exchangeable residual covariance matrix.
3. Removing potential outliers (removed n=12 for weight; n=6 for PA).
4. Only including patients with BMI>25kg/m^2 (analysed n=2298 for weight; n=2159).
5. Adjusting for treatment compliance (including patients in arms A/B that attended at least one intervention session; analysed n=2427 for weight; n=2305 for PA).
6. Adjusting for the delay in intervention start (continuous variable).
7. Adjusting for the unblinding of the research assistant at each follow-up appointment (binary variable).
8. Adjusting for the number of accelerometer wear days at baseline (>3 days, binary variable).
9. Adjusting for the number of accelerometer wear days at baseline (>5 days, binary variable).
10. Adjusting for the number of valid accelerometer wear days at baseline (≥540 minutes, continuous variable). Not completed for weight.
11. Adjusting for the number of accelerometer wear days at each follow-up appointment (continuous variable). Not completed for weight.
12. Adjusting for a BMI score <25 kg/m^2 at baseline (binary variable).
13. Adjusting for a QRisk2 score ≥20.0% at baseline (binary variable).
14. Adjusting for predictors (PHQ-9, smoking status, education) of missing outcome data.

Tables S9-S12 present the output of the primary and sensitivity analyses for each of the primary outcomes, following the numbering above. None of the above sensitivity analyses altered our conclusions for either of the primary outcomes.

The secondary comparison (‘group versus individual’) was assessed on a 5% significance level.

Table S9. Pairwise comparisons of trial arms of treatment effect on PA (steps): primary analysis and sensitivity analyses 1–6.

	**Primary analysis**		**SA1**		**SA2**		**SA3**		**SA4**		**SA5**		**SA6**	
	Estimate (95% CI)	p	Estimate (95% CI)	p	Estimate (95% CI)	p	Estimate (95% CI)	p	Estimate (95% CI)	p	Estimate (95% CI)	p	Estimate (95% CI)	p
12-month follow-up														
Individual – UC	210.22 (-19.46 to 439.91)	0.073	210.01 (-19.67 to 439.69)	0.073	210.04 (-25.62 to 445.71)	0.081	179.85 (-40.31 to 400.01)	0.109	275.19 (25.18 to 525.21)	0.031	196.24 (-55.56 to 448.04)	0.127	374.26 (112.98 to 635.54)	0.005
Group – UC	131.1 (-85.28 to 347.48)	0.24	173.64 (-99.85 to 447.13)	0.213	113.23 (-114.01 to 340.47)	0.33	117.36 (-96.7 to 331.42)	0.283	136.89 (-101.45 to 375.23)	0.26	322.57 (70.19 to 574.95)	0.012	326.57 (78.98 to 574.15)	0.01
Individual – Group	79.12 (-157.73 to 315.98)	0.51	36.37 (-253.49 to 326.23)	0.81	96.82 (-133.93 to 327.57)	0.41	62.49 (-162.36 to 287.33)	0.586	138.3 (-127.42 to 404.02)	0.308	-126.33 (-416.16 to 163.5)	0.393	47.69 (-192.73 to 288.11)	0.697
24-month follow-up														
Individual – UC	7.24 (-224.01 to 238.5)	0.95	7.22 (-223.93 to 238.36)	0.95	2.42 (-235.48 to 240.32)	0.98	9.26 (-217.4 to 235.92)	0.936	-50.78 (-302.04 to 200.47)	0.692	69.37 (-180.85 to 319.58)	0.587	188.76 (-74.39 to 451.91)	0.16
Group – UC	70.05 (-288 to 147.9)	0.53	-24.38 (-299.64 to 250.87)	0.86	-87.82 (-316.3 to 140.65)	0.45	-70.69 (-283.6 to 142.22)	0.515	-34.73 (-276.26 to 206.81)	0.778	11.44 (-238.3 to 261.18)	0.928	102.37 (-145.96 to 350.69)	0.419
Individual – Group	77.29 (-153.37 to 307.95)	0.51	31.6 (-253.97 to 317.18)	0.83	90.25 (-143.39 to 323.88)	0.45	79.95 (-145.24 to 305.14)	0.487	-16.06 (-272.29 to 240.18)	0.902	57.93 (-219.98 to 335.84)	0.683	86.4 (-148.18 to 320.97)	0.47

1. Adjusting for partially nested random effect for therapist.
2. Adding therapist and general practice as random factors and exchangeable residual covariance matrix.
3. Removing potential outliers (removed n=6).
4. Only including patients with BMI>25kg/m^2 (analysed n=2159).
5. Adjusting for treatment compliance (including patients in arms A/B that attended at least one intervention session; analysed n=2305).
6. Adjusting for the delay in intervention start (continuous variable).

Table S10. Pairwise comparisons of trial arms of treatment effect on PA (steps): sensitivity analyses 7-14.

	**SA7**		**SA8**		**SA9**		**SA10**		**SA11**		**SA12**		**SA13**		**SA14**	
	Estimate (95% CI)	p	Estimate (95% CI)	p	Estimate (95% CI)	p	Estimate (95% CI)	p	Estimate (95% CI)	p	Estimate (95% CI)	p	Estimate (95% CI)	p	Estimate (95% CI)	p
12-month follow-up																
Individual – UC	232.51 (-3.13 to 468.15)	0.053	209.9 (-19.93 to 439.72)	0.073	209.24 (-20.7 to 439.17)	0.074	180.32 (-44.47 to 405.11)	0.116	203.39 (-26.09 to 432.87)	0.082	217.25 (-12.85 to 447.35)	0.064	210.97 (-18.8 to 440.74)	0.072	210.17 (-21.76 to 442.09)	0.076
Group – UC	157.32 (-63.04 to 377.68)	0.162	130.84 (-85.62 to 347.3)	0.236	131.51 (-84.97 to 347.98)	0.234	152.17 (-69 to 373.33)	0.177	130.03 (-86.31 to 346.37)	0.239	133.52 (-82.94 to 349.98)	0.227	134.6 (-81.91 to 351.11)	0.223	120.66 (-98.93 to 340.26)	0.281
Individual – Group	75.19 (-163.85 to 314.22)	0.538	79.05 (-157.89 to 316)	0.513	77.73 (-159.22 to 314.68)	0.52	28.15 (-203.26 to 259.57)	0.812	73.36 (-163.2 to 309.92)	0.543	83.73 (-153.41 to 320.86)	0.489	76.37 (-160.73 to 313.47)	0.528	89.5 (-150.65 to 329.66)	0.465
24-month follow-up																
Individual – UC	29.19 (-202.36 to 260.75)	0.805	7.71 (-223.57 to 238.99)	0.948	7.49 (-223.68 to 238.65)	0.949	-11.27 (-252.43 to 229.89)	0.927	-0.03 (-231.34 to 231.28)	1.00	13.61 (-217.34 to 244.55)	0.908	7.62 (-223.7 to 238.95)	0.948	13.43 (-220.15 to 247)	0.91
Group – UC	-63.02 (-281.55 to 155.5)	0.572	-70.76 (-288.72 to 147.2)	0.525	-70.97 (-288.83 to 146.9)	0.523	-44.72 (-272.39 to 182.96)	0.7	-72.71 (-290.64 to 145.22)	0.513	-68.1 (-286.11 to 149.91)	0.54	-66.49 (-284.61 to 151.63)	0.55	-71.61 (-290.8 to 147.58)	0.522
Individual – Group	92.22 (-138.29 to 322.73)	0.433	78.47 (-152.19 to 309.13)	0.505	78.45 (-151.98 to 308.89)	0.505	33.44 (-205.96 to 272.84)	0.784	72.68 (-157.97 to 303.33)	0.537	81.7 (-148.51 to 311.92)	0.487	74.11 (-156.76 to 304.99)	0.529	85.04 (-147.25 to 317.33)	0.473

7. Adjusting for the unblinding of the research assistant at each follow-up appointment (binary variable).
8. Adjusting for the number of accelerometer wear days at baseline (>3 days, binary variable).
9. Adjusting for the number of accelerometer wear days at baseline (>5 days, binary variable).
10. Adjusting for the number of valid accelerometer wear days at baseline (≥540 minutes, continuous variable).
11. Adjusting for the number of accelerometer wear days at each follow-up appointment (continuous variable).
12. Adjusting for a BMI score <25 kg/m^2 at baseline (binary variable).
13. Adjusting for a QRisk2 score ≥20.0% at baseline (binary variable).
14. Adjusting for predictors (PHQ-9, smoking status, education) of missing outcome data.

Table S11. Pairwise comparisons of trial arms of treatment effect on weight (kg): primary analysis and sensitivity analyses 1–6.

	**Primary analysis**		**SA1**		**SA2**		**SA3**		**SA4**		**SA5**		**SA6**	
	Estimate (95% CI)	p	Estimate (95% CI)	p	Estimate (95% CI)	p	Estimate (95% CI)	p	Estimate (95% CI)	p	Estimate (95% CI)	p	Estimate (95% CI)	p
12-month follow-up														
Individual – UC	-0.55 (-0.95 to -0.14)	0.008	-0.55 (-0.95 to -0.14)	0.008	-0.55 (-1.00 to -0.10)	0.017	-0.56 (-0.96 to -0.17)	0.005	-0.55 (-0.96 to -0.15)	0.007	-0.65 (-1.15 to -0.15)	0.011	-0.50 (-0.96 to -0.03)	0.036
Group – UC	-0.52 (-0.90 to -0.13)	0.009	-0.61 (-1.07 to -0.15)	0.009	-0.52 (-0.95 to -0.08)	0.019	-0.53 (-0.90 to -0.16)	0.005	-0.50 (-0.89 to -0.12)	0.01	-0.53 (-1.01 to -0.05)	0.031	-0.41 (-0.87 to 0.04)	0.073
Individual – Group	-0.03 (-0.43 to 0.37)	0.89	0.07 (-0.41 to 0.54)	0.78	-0.03 (-0.47 to 0.41)	0.89	-0.04 (-0.43 to 0.35)	0.85	-0.05 (-0.45 to 0.35)	0.81	-0.12 (-0.61 to 0.37)	0.63	-0.08 (-0.49 to 0.32)	0.687
24-month follow-up														
Individual – UC	-0.42 (-0.93 to 0.09)	0.104	-0.42 (-0.93 to 0.09)	0.103	-0.42 (-0.88 to 0.04)	0.076	-0.47 (-0.96 to 0.02)	0.059	-0.43 (-0.94 to 0.08)	0.099	-0.51 (-1.12 to 0.1)	0.103	-0.38 (-0.94 to 0.18)	0.18
Group – UC	-0.03 (-0.49 to 0.44)	0.91	-0.13 (-0.66 to 0.41)	0.64	-0.01 (-0.45 to 0.43)	0.97	-0.09 (-0.54 to 0.36)	0.69	-0.01 (-0.48 to 0.45)	0.95	0.05 (-0.52 to 0.61)	0.87	0.08 (-0.44 to 0.6)	0.771
Individual – Group	-0.40 (-0.86 to 0.07)	0.096	-0.30 (-0.83 to 0.23)	0.27	-0.41 (-0.85 to 0.04)	0.074	-0.38 (-0.84 to 0.08)	0.11	-0.41 (-0.88 to 0.05)	0.082	-0.56 (-1.11 to 0)	0.05	-0.46 (-0.93 to 0.01)	0.056

1. Adjusting for partially nested random effect for therapist.
2. Adding therapist and general practice as random factors and exchangeable residual covariance matrix.
3. Removing potential outliers (removed n=12).
4. Only including patients with BMI>25kg/m^2 (analysed n=2298).
5. Adjusting for treatment compliance (including patients in arms A/B that attended at least one intervention session; analysed n=2427).
6. Adjusting for the delay in intervention start (continuous variable).

Table S12. Pairwise comparisons of trial arms of treatment effect on weight (kg): sensitivity analyses 7-14.																
	**SA7**		**SA8**		**SA9**		**SA10**		**SA11**		**SA12**		**SA13**		**SA14**	
	Estimate (95% CI)	p	Estimate (95% CI)	p	Estimate (95% CI)	p	Estimate (95% CI)	p	Estimate (95% CI)	p	Estimate (95% CI)	p	Estimate (95% CI)	p	Estimate (95% CI)	p
12-month follow-up																
Individual – UC	-0.02 (-0.86 to 0.82)	0.96	-0.55 (-0.96 to -0.15)	0.007	-0.55 (-0.96 to -0.15)	0.007	--	--	--	--	-0.55 (-0.96 to -0.14)	0.008	-0.55 (-0.95 to -0.14)	0.008	-0.58 (-0.98 to -0.17)	0.006
Group – UC	0.09 (-0.81 to 0.98)	0.85	-0.50 (-0.89 to -0.12)	0.01	-0.51 (-0.9 to -0.12)	0.01	--	--	--	--	-0.52 (-0.91 to -0.13)	0.008	-0.52 (-0.9 to -0.13)	0.009	-0.51 (-0.9 to -0.12)	0.011
Individual – Group	-0.11 (-0.65 to 0.44)	0.71	-0.05 (-0.45 to 0.35)	0.81	-0.04 (-0.44 to 0.36)	0.837	--	--	--	--	-0.03 (-0.43 to 0.37)	0.885	-0.03 (-0.43 to 0.37)	0.887	-0.07 (-0.47 to 0.34)	0.742
24-month follow-up																
Individual – UC	0.1 (-0.83 to 1.02)	0.84	-0.43 (-0.94 to 0.08)	0.099	-0.43 (-0.94 to 0.08)	0.101	--	--	--	--	-0.43 (-0.94 to 0.08)	0.101	-0.42 (-0.93 to 0.09)	0.104	-0.45 (-0.97 to 0.06)	0.083
Group – UC	0.61 (-0.39 to 1.6)	0.23	-0.01 (-0.48 to 0.45)	0.95	-0.02 (-0.48 to 0.45)	0.937	--	--	--	--	-0.03 (-0.5 to 0.44)	0.9	-0.03 (-0.49 to 0.44)	0.914	-0.07 (-0.54 to 0.41)	0.787
Individual – Group	-0.51 (-1.14 to 0.12)	0.11	-0.41 (-0.88 to 0.05)	0.082	-0.41 (-0.87 to 0.06)	0.087	--	--	--	--	-0.4 (-0.86 to 0.07)	0.095	-0.4 (-0.86 to 0.07)	0.095	-0.39 (-0.86 to 0.08)	0.105

7. Adjusting for the unblinding of the research assistant at each follow-up appointment (binary variable).
8. Adjusting for the number of accelerometer wear days at baseline (>3 days, binary variable).
9. Adjusting for the number of accelerometer wear days at baseline (>5 days, binary variable).
10. Adjusting for the number of valid accelerometer wear days at baseline (≥540 minutes, continuous variable). Not completed for weight.
11. Adjusting for the number of accelerometer wear days at each follow-up appointment (continuous variable). Not completed for weight.
12. Adjusting for a BMI score <25 kg/m^2 at baseline (binary variable).
13. Adjusting for a QRisk2 score ≥20.0% at baseline (binary variable).
14. Adjusting for predictors (PHQ-9, smoking status, education) of missing outcome data.

Cost-effectiveness

The number and percentage of participants using specific services or groups of services are shown in Table S13. In the 12-month period prior to baseline assessment the use of all services was very similar between the three treatment arms. There were relatively few people attending A&E or day hospitals or being admitted as inpatients. About half of each group had outpatient contacts and nearly everyone had community contacts. This was particularly due to GP visits.

In the 12 months prior to the 12-month follow-up, around two-thirds of the group arm and three-quarters of the individual arm received the relevant intervention. Visits to A&E and outpatient attendances had increased slightly from baseline but there were no clear differences between treatment arms. These patterns were again observed in the 12-month period prior to the second follow-up, although there was again a slight increase in the proportions having outpatient attendances.

For those with specific service contacts, the average number of contacts is shown in Table S14. At baseline, community services were used more intensely than other services. The inpatient contacts refer to number of days in hospital. It can be seen that there are no major differences between arms. The data for the one-year follow-up show that the individual arm had slightly more intervention contacts than the group arm. Those admitted to hospital from the individual arm had more days in hospital than the other two arms. There were no clear differences between arms in the period up to the 24-month follow-up.

Service costs (including zero costs for non-users) were similar for inpatient care, outpatient attendances, and community contacts (Table S15). Costs of services did not differ markedly between arms, although inpatient costs were somewhat higher for the individual arm at the 12-month follow-up and lower at the 24-month follow-up. The intervention cost was highest for those in the individual arm.

Compared to UC, total costs at baseline were on average £151 more for the group arm (95% CI=–£27 to £328) and £55 more for the individual arm (95% CI=–£96 to £203). The group arm costs were one average £95 more than those for the individual arm (95% CI=–£93 to £299).

After controlling for baseline in a regression model, the total costs in the 12-month period prior to the first follow-up were on average £89 more for the group arm than the UC arm (95% CI=–£274 to £390), and £409 more for the individual arm than the UC arm (95% CI=–£171 to £1133). The individual arm costs were on average £320 more than those for UC (95% CI=–£170 to £1133). By the second follow-up, and again controlling for baseline, the mean costs for the group arm were £82 more than for UC (95% CI=–£93 to £263) and £39 more for UC compared to the individual arm (95% CI=–£108 to £188). Costs were £121 more for the group arm than for the individual arm (95% CI=–£37 to £309).

Mean (SD) total costs over the whole 24-month follow-up period with year two costs discounted by 3.5% were £2071 (£3363) for the group arm, £2230 (£7645) for the individual arm, and £1852 (£3726) for UC. Compared to UC and controlling for baseline, the group arm had costs that were on average £172 higher (95% CI=–£237 to £599) and the individual arm had costs that were £352 higher (95% CI=–£309 to £1271). For cases where both cost and QALY data were available, the group arm had incremental costs of £173 compared to UC and the individual arm incremental costs of £356. These are the incremental costs subsequently used in the incremental cost-effectiveness ratios (ICERs).

Mean EQ-5D tariff scores were similar for each arm and did not change markedly over time (Table S16). Controlling for baseline utility and compared to UC, the group arm resulted in 0.0150 fewer QALYs (95% CI, –0.0388 to 0.0088) and the individual arm in 0.0039 more QALYs (85% CI, –0.0217 to 0.0294). For cases where both cost and QALYs were available, the group arm produced 0.0149 fewer QALYs than UC; and the individual arm 0.0064 more QALYs than UC. These figures are used as the denominator in the ICERs.

Table S13. Number (%) of participants using services in 12 months prior to baseline and each follow-up.

	**Baseline**			**12-month follow-up**			**24-month follow-up**		
	**Group (n=697)**	**Individual (n=523)**	**UC (n=522)**	**Group (n=597)**	**Individual (n=466)**	**UC (n=493)**	**Group (n=570)**	**Individual (n=422)**	**UC (n=459)**
Intervention	0 (0)	0 (0)	0 (0)+	437 (63)	406 (78)	0 (0)	0 (0)	0 (0)	0 (0)

A&E	59 (8)	43 (8)	40 (8)	82 (14)	54 (12)	58 (12)	80 (14)	67 (16)	64 (14)
Day hospital	55 (8)	42 (8)	42 (8)	40 (7)	36 (8)	30 (6)	26 (5)	18 (4)	12 (3)
Inpatient	43 (6)	31 (6)	20 (4)	47 (8)	27 (6)	31 (6)	37 (6)	24 (6)	29 (6)
Outpatient	355 (51)	268 (51)	266 (51)	346 (58)	280 (60)	301 (61)	383 (67)	272 (65)	302 (66)
Community	667 (96)	502 (96)	503 (96)	588 (98)	461 (99)	489 (99)	564 (99)	412 (98)	449 (98)

Table S14. Mean (SD) number of contacts by those using services in 12 months prior to baseline and each follow-up.

	**Baseline**			**12-month follow-up**			**24-month follow-up**		
	**Group (n=697)**	**Individual (n=523)**	**UC (n=522)**	**Group (n=597)**	**Individual (n=466)**	**UC (n=493)**	**Group (n=570)**	**Individual (n=422)**	**UC (n=459)**
Intervention	-	-	-	7.6 (3.0)	8.8 (3.4)	-	-	-	-
A&E	1.5 (1.6)	1.3 (0.6)	1.3 (0.7)	1.5 (1.4)	1.3 (1.1)	1.5 (1.0)	1.8 (3.3)	1.3 (0.8)	1.6 (1.7)
Day hospital	3.4 (4.1)	1.8 (2.3)	2.4 (2.0)	1.8 (1.4)	1.7 (1.9)	1.6 (1.2)	1.5 (1.0)	1.7 (1.5)	1.0 (0.0)
Inpatient	4.7 (10.1)	4.7 (5.0)	3.8 (5.9)	7.4 (12.4)	14.7 (47.9)	6.9 (21.1)	5.8 (9.3)	3.0 (3.1)	4.0 (3.3)
Outpatient	3.2 (4.2)	3.0 (4.1)	3.2 (4.5)	3.6 (4.2)	4.1 (4.5)	4.0 (5.4)	3.9 (4.2)	4.2 (4.6)	4.1 (4.0)
Community	7.1 (5.4)	7.5 (8.0)	7.0 (6.9)	8.8 (6.5)	9.2 (7.1)	9.1 (6.5)	9.4 (8.1)	8.9 (7.3)	9.2 (6.8)

Table S15. Mean (SD) service costs (2015/16 £s) in 12 months prior to baseline and each follow-up.

	**Baseline**			**12-month follow-up**			**24-month follow-up**		
	**Group (n=697)**	**Individual (n=523)**	**UC (n=522)**	**Group (n=697)**	**Individual (n=523)**	**UC (n=522)**	**Group (n=697)**	**Individual (n=523)**	**UC (n=522)**
Intervention	0 (0)	0 (0)	0 (0)+	55 (50)	136 (98)	0 (0)	0 (0)	0 (0)	0 (0)+
A&E	19 (94)	16 (58)	15 (59)	30 (107)	23 (83)	27 (90)	36 (204)	32 (88)	33 (123)
Day hospital	193 (1049)	105 (573)	137 (618)	85 (405)	95 (497)	68 (341)	49 (271)	53 (328)	19 (114)
Inpatient	172 (1605)	164 (955)	85 (788)	340 (2333)	499 (6940)	256 (3207)	219 (1609)	99 (590)	149 (745)

Outpatient	222 (460)	210 (442)	221 (485)	284 (492)	327 (542)	329 (630)	355 (524)	366 (568)	361 (505)
Community	225 (260)	241 (331)	222 (187)	306 (253)	317 (233)	306 (220)	324 (253)	306 (232)	321 (279)
**Total**	**831 (2127)**	**736 (1299)**	**680 (1132)**	**1107 (2520)**	**1407 (7067)**	**985 (3312)**	**984 (1915)**	**855 (1079)**	**884 (1114)**
We could not calculate the cost of lost employment as these data were not adequately recorded.									

Table S16. Mean (SD) EQ-5D tariffs scores by arm and time point.

	**Group**	**Individual**	**UC**
Baseline	0.8738 (0.1973)	0.8831 (0.1870)	0.8828 (0.1630)
12-month follow-up	0.8869 (0.1958)	0.9085 (0.1632)	0.8947 (0.1688)
24-month follow-up	0.8994 (0.1639)	0.9064 (0.1475)	0.9144 (0.1459)
QALYs	1.7717 (0.3073)	1.8123 (0.2306)	1.8014 (0.2462)

The group arm was less effective than UC and more expensive. As such it was *dominated*. Individual was more expensive and more effective. The ICER was £55,625 per QALY (£356 divided by 0.0064 QALYs). Uncertainty around the estimates are shown in the cost-effectiveness planes (Figures S2 and S3). In Figure SXX, comparing the group arm to UC, it can be clearly seen that the majority (69%) of bootstrapped replications fall in the North-West where the group arm has higher costs and produces fewer QALYs. 8.5% fall in the North-East quadrant where costs are still higher but more QALYs are produced. In the South-East quadrant where costs are lower and more QALYs are produced there are 4% of replications. Finally, there are 18.5% of replications in the South-West quadrant where the group arm is less expensive but produces fewer QALYs.

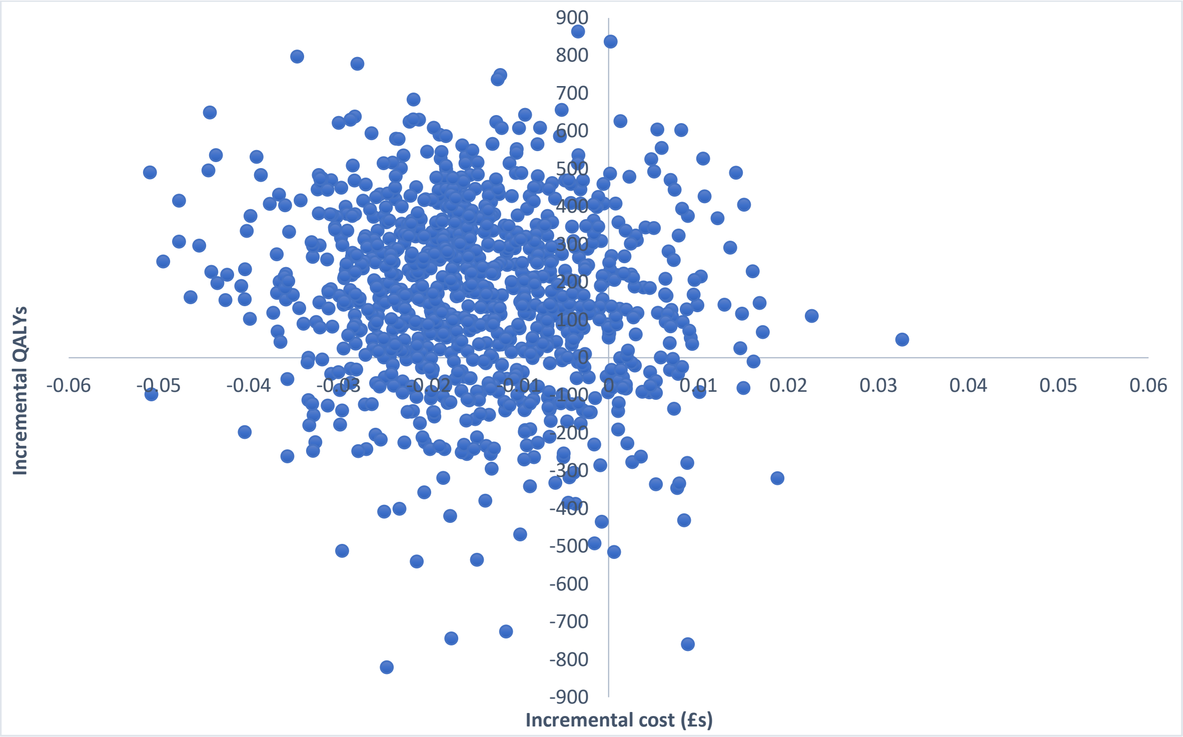

Figure S2. Cost-effectiveness plane comparing the group arm and UC.

In Figure S3, comparing the individual arm to UC, 52.3% of replications are in the North-East quadrant where the individual arm has higher costs and produces more QALYs, 15.7% are in the South-East quadrant where costs are lower and there are more QALYs produced, 5.8% are in the South-West quadrant where costs are lower and there are fewer QALYs produced, and finally there are 26.2% of replications in the North-West quadrant where the individual arm has higher costs and produces fewer QALYs.

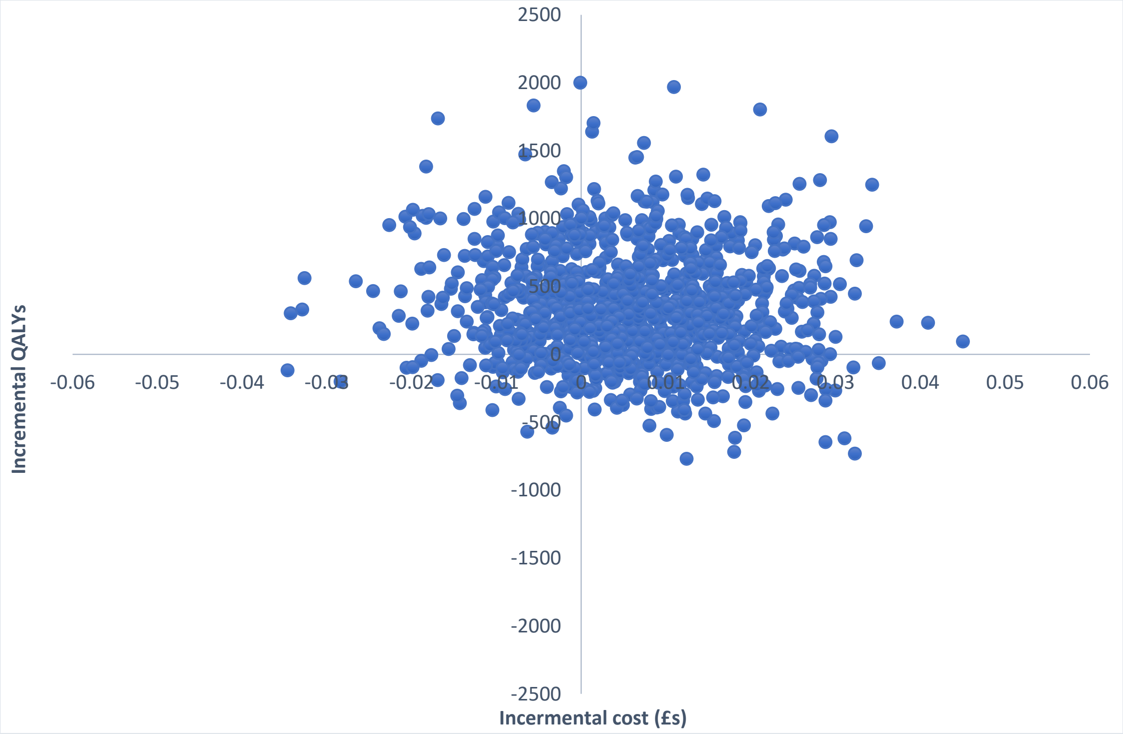

Figure S3. Cost-effectiveness plane comparing the individual arm and UC.

Figure S4 shows the cost-effectiveness acceptability curves where all three arms are compared for different values placed on a QALY gain. When a zero value is placed on a QALY, UC has by far the highest probability of being the most cost-effective option (in this situation, only costs are relevant and UC is less expensive). As the value on a QALY is increased, the probability that the individual arm is the most cost-effective option increases steadily while the other two arms see a fall in the probability that they are the most cost-effective. This is to be expected as the

individual arm is more expensive and more effective than the other options and as the effect (i.e. increased QALYs) is valued more it increasingly offsets the cost. However, at a value of £30,000 (above which NICE is likely to decide an intervention is not cost-effective) the individual arm has a 37.4% likelihood of being the most cost-effective option compared to 58.1% for UC. At this value, the group arm has a likelihood of 4.5% of being the most cost-effective option.

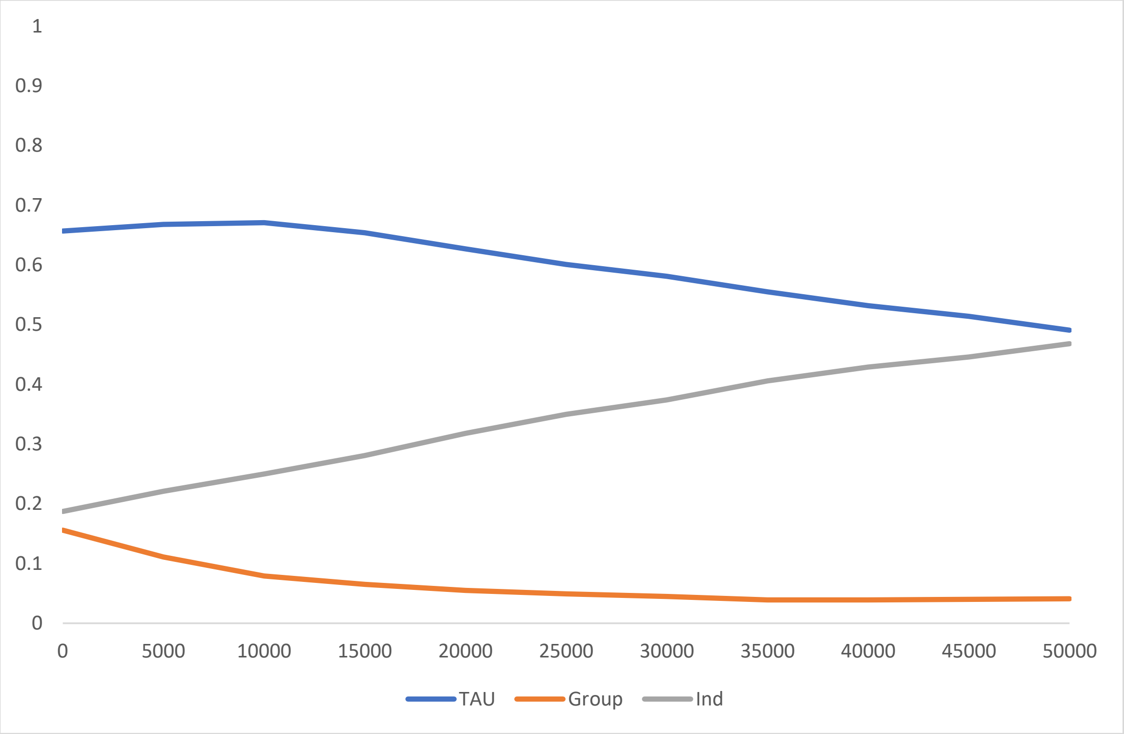

Figure S4. Cost-effectiveness acceptability curves.

In sensitivity analyses, the intervention costs were increased and decreased by 25% and 50% respectively to reflect different staff grades delivering the interventions. When intervention costs were reduced by 25%, the group arm had costs that were on average £188 more than for UC while costs for the individual arm were £395 more than for UC. The differences when intervention costs were increased by 50% were £204 and £434 respectively. Not surprisingly

these increased differences mean that the interventions are even less likely to be cost-effective compared to UC.

When intervention costs are reduced by 25%, the group arm has costs that are on average £157 more than for UC. With fewer QALYs, UC is still dominant. Individual is now £317 more than for UC resulting in a cost per QALY of £49,531. With a reduction of 50%, the group arm still has higher costs than UC (by £141) and so continues to be dominated while the individual arm has costs that are £278 higher resulting a cost per QALY of £43,438.

Adverse events

Table S17. Summary of adverse events by subcategory and trial arm

	Trial arm						
	Group (n=697)		Individual (n=523)		UC (n=522)		
	n	%	n	%	n	%	χ^2-test output[a]
Any AE reported	212	30.4	151	28.9	160	30.7	0.48
Multiple AEs	34	4.9	17	3.3	23	4.4	4.20
Two AEs	27	3.9	16	3.1	22	4.2	
Three AEs	7	1.0	1	0.2	1	0.2	
AE type							
Death	3	0.4	2	0.4	4	0.8	0.92
Any physical injury	109	15.6	65	12.4	85	16.3	3.61
Dislocation	5	0.7	1	0.2	2	0.4	1.96
Fracture	16	2.3	8	1.5	7	1.3	1.82
Sprain/strain	10	1.4	17	3.3	12	2.3	3.90
Muscle/tendon	28	4.0	16	3.1	25	4.8	2.33
Other	48	6.9	22	4.2	39	7.5	5.72
Fall	37	5.3	17	3.3	23	4.4	3.00
Any cardiovascular event	42	6.0	22	4.2	22	4.2	2.94[b]
Angina	2	0.3	2	0.4	1	0.2	0.33
Atrial fibrillation	7	1.0	2	0.4	4	0.8	1.56
CHD	0	0.0	0	0.0	1	0.2	2.34
Coronary bypass	4	0.6	0	0.0	0	0.0	6.01[c]
Myocardial infarction	6	0.9	3	0.6	3	0.6	0.50
Stroke/TIA	9	1.3	3	0.6	6	1.1	1.60
Other	14	2.0	15	2.9	7	1.3	3.03

[a] χ^2-tests, two degrees of freedom and p>0.05, except where indicated

[b]degrees of freedom=8
[c]p=0.0495

REFERENCES

1 Catellier DJ, Hannan PJ, Murray DM, *et al.* Imputation of missing data when measuring physical activity by accelerometry. *Med Sci Sport Exerc* 2005; **37**: S555-62.

2 Ae Lee J, Gill J. Missing value imputation for physical activity data measured by accelerometer. *Stat Methods Med Res* 2018; **27**: 490–506.

3 Staudenmayer J, Zhu W, Catellier DJ. Statistical considerations in the analysis of accelerometry-based activity monitor data. *Med Sci Sports Exerc* 2012; **44**: S61-7.
